# *Split Feeding* for semi-heavy laying hens from 105 to 120 weeks of age: Performance, egg quality, biochemical parameters, reproductive tract morphometry and bone quality

**DOI:** 10.1016/j.psj.2026.106917

**Published:** 2026-04-09

**Authors:** Aline Beatriz Rodrigues, Adiel Vieira de Lima, Paloma Eduarda Lopes de Souza, Carlos Henrique do Nascimento, Raiane dos Santos Silva, Raul da Cunha Lima Neto, Matheus Ramalho de Lima, Apolônio Gomes Ribeiro, Edijanio Galdino da Silva, Ariolino Moura de Oliveira Neto, Marcos Rafael de Sousa Rodrigues, José de Arimatéia de Freitas Pinto, Ricardo Romão Guerra, Lucas Rannier Ribeiro Antonino Carvalho, Fernando Guilherme Perazzo Costa

**Affiliations:** a Department of Animal Science, Federal University of Paraíba, Areia, Paraíba, Brazil; b Federal University of Western Pará, Institute of Biodiversity and Forests, Santarém, PA, Brazil; c Federal Rural University of the Semi-Arid, Department of Animal Science, Mossoró, RN, Brazil; d Department of Veterinary Sciences, Federal University of Paraíba, Areia, Paraíba, Brazil; e Nutrivet Animal Nutrition, São Carlos, SP, Brazil; f Department of Physiology and Pharmacology, Karolinska Institutet, Biomedicum 5B, Solnavägen 9, S-171 77, Stockholm, Sweden

**Keywords:** Calcium, Feed efficiency, Eggshell quality

## Abstract

This study evaluated different intensities of *split feeding* on performance, egg quality, oviposition time, serum biochemical parameters, reproductive tract morphometry, and bone quality of semi-heavy laying hens in the late production phase. A total of 160 Novogen Brown hens (105 weeks of age) were assigned to a completely randomized design with four treatments and five replicates of eight birds. Treatments included: CONTROL, with a standard diet offered twice daily; LIGHT, with a morning diet containing 5% higher crude protein (CP) and metabolizable energy (ME) and 40% lower calcium (Ca), and an afternoon diet with opposite adjustments; MODERATE, with +8% CP, +10% ME, and −50% Ca in the morning and inverse levels in the afternoon; and INTENSE, with +11% CP, +15% ME, and −60% Ca in the morning and inverse levels in the afternoon, relative to the control diet. The experimental period lasted 105 days, and diets were formulated to synchronize nutrient supply with daily physiological demands. No significant differences were observed among treatments for egg production, egg weight, or egg mass, indicating maintenance of productive performance. *Split feeding* improved egg quality, increasing eggshell strength, Haugh units, and yolk index, especially in the moderate and intense treatments. Oviposition was more concentrated during early hours of the day, facilitating egg collection. The moderate treatment resulted in lower serum urea concentrations and adequate cholesterol and triglyceride levels, indicating improved protein utilization and metabolic balance. Reproductive tract morphometry showed increased height and area of magnum and uterine folds in hens subjected to *split feeding*. Bone quality, assessed by the Seedor index, was reduced in higher-intensity treatments, indicating greater calcium mobilization without compromising bone mechanical strength. In conclusion, moderate *split feeding* was the most effective strategy for semi-heavy laying hens between 105 and 120 weeks of age, improving nutritional synchronization, metabolic efficiency, reproductive tract integrity, and egg quality without impairing productive performance.

## Introduction

Commercial laying hens have a high capacity to maintain egg production over long periods; however, advancing age is associated with a gradual decline in laying rate and egg quality, particularly eggshell quality, especially in brown layers. These effects become more evident in hens older than 100 weeks of age, when further deterioration in eggshell integrity, along with increased bone fragility, begins to compromise productivity and flock longevity, representing a major challenge for commercial egg production systems (Carvalho et al., 2013; Nak et al., 2015; Reis et al., 2020).

Egg formation occurs in a continuous cycle of approximately 24 to 26 hours, involving sequential events in different segments of the oviduct. Albumen deposition occurs predominantly in the magnum, whereas eggshell mineralization takes place in the uterus during the final stages of the cycle, mainly at night (Santos et al., 2017). Consequently, the nutritional requirements of laying hens vary throughout the day in response to circadian rhythms that regulate the different stages of egg formation.

Calcium plays a central role in this process, as it is the main inorganic component of the eggshell and participates in several metabolic functions. During eggshell formation, calcium demand increases markedly, with part of this mineral being supplied through mobilization of medullary bone reserves (Whitehead, 2004; Fleming et al., 2006). In older laying hens, prolonged reliance on this mobilization may intensify bone mass loss and compromise eggshell quality, especially when dietary supply is not adequately synchronized with daily physiological demands (Ito et al., 2006).

In addition to changes in mineral metabolism, aging is also associated with structural and functional alterations in the reproductive tract, particularly in the magnum and uterus, organs essential for the deposition of egg components. These alterations may reduce secretory activity and impair the efficiency of albumen and eggshell formation, directly affecting egg quality (Artoni et al., 2019; Hosotani et al., 2023). In this context, precision nutritional strategies such as split-feeding have been proposed to better align nutrient supply with the physiological demands of hens throughout the day. While the classical approach involves higher protein and amino acid supply in the morning to support albumen synthesis, alternative strategies have also been investigated, including increased protein and amino acid levels in the afternoon diets to compensate for lower morning intake or to ensure adequate daily amino acid supply (Horváth et al., 2024). Despite its relevance, studies relating precision nutritional strategies to morphological parameters of the reproductive tract in older laying hens remain limited.

In this context, the *split-feeding* method has been proposed as a nutritional strategy capable of aligning nutrient supply with the daily physiological rhythm of egg formation. This approach consists of providing diets with higher energy and protein density in the morning, followed by diets with higher calcium content and adjusted phosphorus levels in the afternoon, aiming to meet the increased mineral demand associated with nighttime eggshell formation (Molnár et al., 2018). Although increasing dietary calcium in conventional feeding programs or providing calcium supplementation in the afternoon may improve eggshell quality, these strategies do not fully account for the temporal variation in nutrient requirements, which is more precisely addressed by split-feeding systems.

Although studies indicate that split feeding may improve nutritional efficiency and eggshell quality, most available research has been conducted with young laying hens or during early production phases, with limited information regarding its effects on semi-heavy and older hens. Therefore, the objective of this study was to evaluate the effects of the *split-feeding* method in semi-heavy laying hens aged between 105 and 120 weeks, assessing its impact on productive performance, egg quality, oviposition time, serum biochemical parameters, magnum and uterine morphometry, and bone quality, compared with a conventional single-diet feeding program.

## Materials and methods

The experimental trial was conducted at the Poultry Research Unit of the Department of Animal Science, Center for Agricultural Sciences, Federal University of Paraíba (CCA/UFPB), in Areia, Paraíba, Brazil. The experimental protocol No. 5262050226 was approved by the Animal Use Ethics Committee of the Federal University of Paraíba (CEUA-UFPB).

### Animals, facilities, and experimental design

A total of 160 semi-heavy laying hens of the Novogen Brown strain, with an initial age of 105 weeks, were used. The birds were obtained from a commercial flock (Ovo Novo farm, Pernambuco, Brazil) and were selected based on uniform body weight and laying performance at the beginning of the experimental period. The birds were distributed into four treatments with five replicates, totaling 20 experimental units, each consisting of eight hens. A completely randomized experimental design was adopted.

The experiment lasted 105 days and was divided into five periods of 21 days each. The birds were housed in a conventional poultry house with natural ventilation and no active environmental control. Climatic conditions during the experimental period were characterized based on data from the meteorological station of the National Institute of Meteorology – INMET (INMET, 2025), obtained through the Meteorological Database for Teaching and Research (BDMEP), which recorded an average temperature of 23.4°C and an average relative humidity of 82.9%.

The lighting program consisted of natural daylight supplemented with artificial lighting, which was turned on daily from 17:00 to 22:00 h to extend the natural photoperiod, ensuring a total photoperiod of approximately 16 hours of light per day throughout the experimental period.

### Experimental diets

The diets were formulated with different levels of metabolizable energy (ME), crude protein (CP), and calcium (Ca) and were offered at two distinct times of the day, in the morning and in the afternoon, according to the experimental treatments. Diet formulation was based on the nutritional requirements recommended by the Brazilian Tables for Poultry and Swine (Rostagno et al., 2024).

**Fig. 1:**
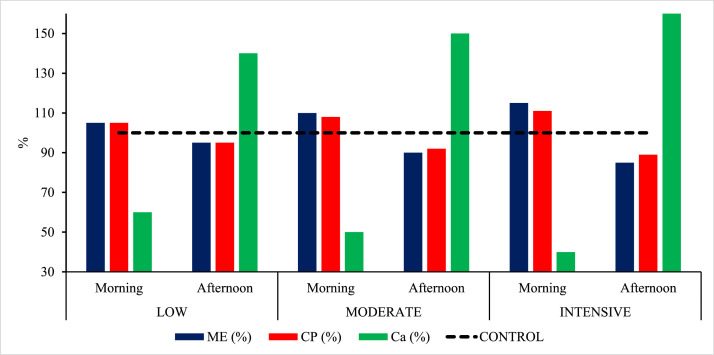
Variation in metabolizable energy (ME), crude protein (CP), and calcium (Ca) levels in the experimental diets. CONTROL: Standard diet provided twice daily, formulated with 2,800 kcal/kg ME, 15.51% CP, and 4.55% Ca. LOW: Morning diet containing 5% more CP and ME and 40% less Ca than the control diet, and afternoon diet containing 5% less CP and ME and 40% more Ca. MODERATE: Morning diet containing 8% more CP, 10% more ME, and 50% less Ca, and afternoon diet containing 8% less CP, 10% less ME, and 50% more Ca. INTENSIVE: Morning diet containing 11% more CP, 15% more ME, and 60% less Ca, and afternoon diet containing 11% less CP, 15% less ME, and 60% more Ca.

The treatments were established as follows: CONTROL – standard diet offered twice a day, formulated with 2,800 kcal/kg of metabolizable energy (ME), 15.51% crude protein (CP), and 4.55% calcium (Ca); MILD (mild split feeding) – morning diet containing 5% more CP and ME and 40% less Ca compared with the control diet, and afternoon diet containing 5% less CP and ME and 40% more Ca; MODERATE (moderate split feeding) – morning diet containing 8% more CP, 10% more ME, and 50% less Ca, and afternoon diet containing 8% less CP, 10% less ME, and 50% more Ca; and INTENSE (intense split feeding) – morning diet containing 11% more CP, 15% more ME, and 60% less Ca, and afternoon diet containing 11% less CP, 15% less ME, and 60% more Ca. The variation in nutritional levels among treatments is presented in Fig. 1, while the percentage composition of ingredients and the chemical composition of the diets are shown in Table 1.

**Table 1:** Experimental diets and chemical composition.

**Ingredients**	**CONTROL**	**LOW**		**MODERATE**		**INTENSIVE**	
	**Morning/** **Afternoon**	**Morning**	**Afternoon**	**Morning**	**Afternoon**	**Morning**	**Afternoon**
Corn 7,88%	649.7	678.2	621.7	712.5	555.3	687.3	491.2
Wheat bran	35.0	47.0	24.0	0.0	98.0	0.0	178.0
Soybean meal 45,22%	191.0	200.0	181.0	217.0	161.0	233.0	133.0
Soybean oil	0.0	0.0	0.0	7.5	0.0	29.1	0.0
Limestone 37/37	116.5	67.0	165.5	55.0	178.0	42.5	190.0
Salt	3.75	3.74	3.77	3.75	3.75	3.75	3.74
TRIC CEZ 0.05% PC^1^	0.50	0.50	0.50	0.50	0.50	0.50	0.50
DL-Methionine 99%	1.05	1.09	1.00	1.20	0.91	1.31	0.86
L-Lysine HCl 78%	0.00	0.00	0.02	0.03	0.02	0.00	0.25
Choline chloride 60%	0.50	0.50	0.50	0.50	0.50	0.50	0.50
Vitamin–mineral premix²	2.00	2.00	2.00	2.00	2.00	2.00	2.00
**TOTAL**	**1,000**	**1,000**	**1,000**	**1,000**	**1,000**	**1,000**	**1,000**
ME kcal/kg	2,800	2,940	2,660	3,080	2,520	3,220	2,380
CP %	15.51	16.33	14.66	16.70	14.32	17.24	13.75
DCP %	13.09	13.81	12.34	14.21	11.94	14.69	11.29
Dig Arg %	0.91	0.96	0.85	0.98	0.84	1.02	0.80
Dig Lys %	0.70	0.73	0.66	0.77	0.63	0.81	0.60
Dig Met %	0.33	0.35	0.31	0.36	0.30	0.38	0.28
Dig Met + Cys %	0.57	0.59	0.54	0.61	0.52	0.64	0.49
Dig Thr %	0.55	0.58	0.53	0.60	0.50	0.62	0.47
Dig Trp %	0.17	0.17	0.16	0.18	0.15	0.19	0.15
Dig Ile %	0.58	0.61	0.55	0.63	0.52	0.66	0.48
EE %	2.96	3.12	2.79	3.88	2.75	5.95	2.73
LA %	1.45	1.53	1.37	5.39	1.34	16.38	1.32
CF (%)	2.22	2.42	2.03	2.12	2.51	2.14	3.01
Starch (%)	44.05	46.29	41.83	47.21	39.64	45.67	37.70
Ash (%)	13.44	8.74	18.13	7.47	19.46	6.32	20.78
Ca (%)	4.55	2.73	6.37	2.28	6.83	1.82	7.28
P Total %	0.66	0.68	0.63	0.65	0.68	0.66	0.72
AvP %	0.45	0.46	0.45	0.45	0.47	0.45	0.50
DP %	0.46	0.47	0.45	0.45	0.47	0.45	0.49
Na %	0.17	0.17	0.17	0.17	0.17	0.17	0.17
Cl %	0.32	0.32	0.32	0.32	0.31	0.32	0.32
Ca/AvP	10.01	5.89	14.31	5.10	14.40	4.06	14.45

1 Minimum guaranteed levels: Phytase – 1,800,000 FTU/kg. ² Provided per kilogram of diet: 66 mg Fe (FeSO₄·7H₂O), 83 mg Zn (ZnSO₄·7H₂O), 80 mg Mn (MnSO₄·H₂O), 1 mg I (KI), and 6.8 mg Cu (CuSO₄·5H₂O); 11,700 IU vitamin A; 3,600 IU vitamin D₃; 21 IU vitamin E; 4.2 mg vitamin K₃; 3.0 mg vitamin B₁; 10.2 mg vitamin B₂; 0.9 mg folic acid; 15 mg calcium pantothenate; 45 mg niacin; 5.4 mg vitamin B₆; 24 μg vitamin B₁₂; and 150 μg biotin. ME: Metabolizable energy; CP: Crude protein; DCP: Digestible crude protein; Dig Arg: Digestible arginine; Dig Lys: Digestible lysine; Dig Met: Digestible methionine; Dig Met + Cys: Digestible methionine + cysteine; Dig Thr: Digestible threonine; Dig Trp: Digestible tryptophan; Dig Ile: Digestible isoleucine; EE: Ether extract; LA: Linoleic acid; CF: Crude fiber; Ca: Calcium; Total P: Total phosphorus; AvP: Available phosphorus; Dig P: Digestible phosphorus; Na: Sodium; Cl: Chloride; Ca/AvP: Calcium-to-available phosphorus ratio. CONTROL: Standard diet provided twice daily, formulated with 2,800 kcal/kg ME, 15.51% CP, and 4.55% Ca. LOW: Morning diet containing 5% more CP and ME and 40% less Ca than the control diet, and afternoon diet containing 5% less CP and ME and 40% more Ca. MODERATE: Morning diet containing 8% more CP, 10% more ME, and 50% less Ca, and afternoon diet containing 8% less CP, 10% less ME, and 50% more Ca. INTENSIVE: Morning diet containing 11% more CP, 15% more ME, and 60% less Ca, and afternoon diet containing 11% less CP, 15% less ME, and 60% more Ca.

### Dietary supply and analyses

The feed was offered to the birds at two times during the day: in the morning at 07:00 h and in the afternoon at 16:00 h. Each cage was equipped with two feeders, which were alternated at feeding times, allowing the specific diets to be supplied according to the experimental treatment at the appropriate time. Productive performance, egg quality, oviposition time, serum biochemical parameters, histology of the reproductive tract, and bone quality were evaluated.

### Zootechnical variables

Bird performance was evaluated based on egg production (%), calculated as the ratio between the number of eggs produced and the number of housed birds, multiplied by 100; egg weight (g); feed intake (g/bird/day); feed conversion per dozen eggs (kg/dozen); feed conversion per egg mass (kg/kg); and the percentage of dirty or cracked eggs.

Feed intake was determined as the difference between the amount of feed offered at the beginning of each experimental period and the weight of the leftovers at the end of the period. Values were corrected for mortality, when applicable, to obtain actual feed intake. The diets supplied in the morning and afternoon were stored in separate containers, allowing individual control of each feeding. At the end of each experimental period, feed leftovers were weighed to determine the intake of each diet.

Feed conversion per dozen eggs was calculated as the ratio between feed intake and the number of dozens of eggs produced, with values corrected for bird mortality. To determine average egg weight, during the last three days of each experimental period, all eggs from each experimental unit were collected and individually weighed using an analytical balance with a precision of four decimal places (0.0001 g).

Egg mass was calculated by multiplying the number of eggs produced by the average egg weight of each replicate within the period. Feed conversion per egg mass (kg feed/kg eggs) was determined as the ratio between feed intake and the egg mass produced per replicate in each experimental period.

The occurrence of dirty and cracked eggs was recorded daily and expressed as a percentage of the total number of eggs produced per experimental unit.

### Egg quality and oviposition time

During the last three days of each experimental cycle, egg quality evaluations were performed using two eggs per replicate. The variables analyzed included egg weight (g), specific gravity (g/cm³), percentages of yolk, albumen, and shell, shell thickness (mm), yolk color, Haugh unit, shell strength (kg), and yolk index.

Egg weight was determined by individual weighing on an analytical balance with a precision of four decimal places (0.0001 g). Specific gravity was determined according to the methodology described by Freitas et al. (2004), based on Archimedes’ principle, in which specific gravity was calculated as the ratio between egg weight in air and the weight of the displaced water when the egg was fully submerged, with correction for water temperature. Yolk color was evaluated using the DSM YolkFan™ color fan (scale from 1 to 15). Albumen quality was determined by the Haugh unit, in which eggs were broken onto a flat glass surface and the height of the thick albumen (mm) was measured using a depth micrometer. Albumen height and egg weight values were applied to the following equation: HU = 100 × log (H − 1.7 × P^0.37^ + 7.6), where HU = Haugh unit, H = albumen height (mm), and P = egg weight (g).

Subsequently, the yolk was separated from the albumen and weighed on a precision balance (0.01 g), and its percentage was obtained by dividing yolk weight by egg weight and multiplying by 100. Albumen percentage was obtained using the following equation: % albumen = 100 − (% yolk + % shell). Albumen weight was determined as the difference between egg weight and the sum of yolk and shell weights. Eggshells were identified, dried in a forced-air oven at 55–60°C for 24 h, and subsequently weighed on a digital balance with a precision of 0.0001 g to obtain the mean shell weight. Shell percentage was calculated as the ratio between mean shell weight and mean egg weight, multiplied by 100.

For shell thickness determination, fragments were taken from the equatorial region of the eggs, and measurements were performed using a digital micrometer (Mitutoyo, 0–25 mm, precision of 0.001 mm; Kawasaki, Japan), with the final thickness considered as the average of three regions of the egg. Shell strength was evaluated by subjecting the egg to increasing pressure applied by a digital hardness tester IPGW-02 Impac© (São Paulo, Brazil) in the longitudinal direction. The yolk index was calculated as the ratio between yolk height and yolk width.

Oviposition time was evaluated every 15 days throughout the experimental period. For this purpose, the number of eggs produced in each experimental unit was recorded at two-hour intervals, from 06:00 to 18:00 h, considering the time at which oviposition occurred.

### Serum biochemical analyses

At the end of the experimental cycle (fifth period), before sample collection, the birds were subjected to a 6-h fasting period. Blood samples (approximately 4 mL) were collected from five birds per treatment by jugular vein puncture using sterile 13 × 0.4 mm needles. Blood was collected into dry tubes containing a clot activator (BD Vacutainer® Dry). The samples were allowed to stand at room temperature for 30 min to allow clot formation and were then centrifuged at 3,500 rpm for 10 min in a bench centrifuge (SL-702/RAF30, Solab, Piracicaba, SP, Brazil) to obtain the serum.

The separated serum was transferred to 2-mL Eppendorf microtubes and stored frozen until analysis. Serum biochemical analyses were performed at the Poultry Science Laboratory of the Federal University of Paraíba using commercial diagnostic kits (Labtest Diagnóstica S.A.®). The evaluated parameters included glucose (GLU; Ref. 133), triglycerides (TG; Ref. 87), cholesterol (CHOL; Ref. 1082), uric acid (UA; Ref. 140), urea (U; Ref. 1013), creatinine (CRE; Ref. 1010), aspartate aminotransferase (AST; Ref. 1009), alanine aminotransferase (ALT; Ref. 1008), gamma-glutamyl transferase (GGT; Ref. 1058), alkaline phosphatase (ALP; Ref. 1011), total protein (TP; Ref. 1085), albumin (ALB; Ref. 1007), calcium (Ca; Ref. 1084), and phosphorus (P; Ref., 12-200). All analyses were performed using an automatic biochemical analyzer (Sinnowa SX-260® model, Nanjing, China).

### Histomorphometric analyses

For reproductive system morphometry (magnum and uterus), fragments of the magnum (1 cm of the organ per bird) and uterus (1 cm² from the middle region per bird) were collected from eight birds per treatment at the end of the experimental period. The material was fixed in 10% buffered formalin in phosphate-buffered saline (PBS), pH 7.2, for 24 h, according to the methodology described by Junqueira and Junqueira (1983). Samples were processed using routine histological procedures, and transverse histological sections of 5 µm thickness were obtained and stained with the Periodic Acid–Schiff histochemical reaction associated with hematoxylin (PAS–hematoxylin; PAS-H). Slides were examined under a bright-field light microscope (Olympus BX53F, Tokyo, Japan) coupled to an Olympus DP73 digital camera, and digital images were captured and analyzed using cellSens Dimension® software.

For each variable analyzed, six measurements per section and per bird were performed (Jiang et al., 2021) using the 4 × objective, totaling 48 observations per variable per treatment. The variables evaluated were fold height (µm), fold width (µm), and fold area (µm²). Fold height was measured from the mucosa to the apical epithelium, considering only intact folds. Fold width was measured at three distinct points of each fold, and the final value was obtained as the mean of these measurements. Fold area was determined by tracing the complete contour of each fold, delimiting its entire epithelial and luminal extension using the image analysis software.

### Bone quality analysis

At the end of the experiment, eight birds from each treatment were euthanized for the collection of materials for subsequent analyses. Tibiae and femurs from all birds were collected and carefully defleshed for the evaluation of bone strength, Seedor index (mm), and mineral matter (%). The determination of the Seedor index followed the methodology described by Seedor et al. (1991). The Seedor index, which represents an indicator of bone density, was obtained by weighing the tibiae and femurs on a semi-analytical digital scale (0.01 g) and measuring bone length using a digital caliper. The formula used was: Seedor index = bone weight (mg) / bone length (mm). Bone strength was determined using a universal texture analyzer (TA. XT Plus, Stable Micro Systems, Surrey, UK), applying a load of 50 kg at a speed of 50 mm/min. For the determination of mineral matter content, tibiae and femurs were previously lyophilized for 72 h under vacuum at −50°C, aiming to remove moisture without altering the mineral composition of the bone tissue, as described by Galia et al. (2009). After lyophilization, the bones were ground and subjected to incineration in a muffle furnace at 550°C until complete combustion of organic matter, and the resulting residue was considered ash, corresponding to total mineral matter (%), according to the methodology described by Silva and Queiroz (2002).

### Statistical analysis

Statistical analyses were performed using SAS (2011). The results were subjected to the Shapiro–Wilk test to verify normality. Subsequently, the data were analyzed by ANOVA, and treatment means were compared using Tukey’s test at a 5% probability level.

## Results and discussion

Regarding productive performance (Table 2), no significant differences were observed for egg production, total feed intake, egg mass, feed conversion per egg mass, or feed conversion per dozen eggs, indicating that variation in the timing of diet supply did not compromise the daily energy and protein balance of the hens. The absence of a significant effect on performance can be attributed to the natural ability of laying hens to select diets that meet their physiological requirements, demonstrating selective feeding behavior when offered diets with different nutrient compositions. This mechanism allows birds to balance their intake and optimize nutrient utilization throughout the egg formation cycle, maintaining homeostasis (Rech and Lora, 2022).

**Table 2:** Performance of semi-heavy laying hens subjected to the *split-feeding* method.

Variables*	**CONTROL**	**LOW**	**MODERATE**	**INTENSIVE**	***P-Value***	**SEM**
EP %	67.2	73.0	67.50	68.8	0.250	1.169
MFI g/day	53.5b	53.8ab	56.5ab	58.0a	0.026	0.660
AFI g/day	55.7a	53.1ab	51.5ab	49.9b	0.009	0.694
TFI g/day	109.2	106.9	107.9	108.0	0.796	0.779
EW (g)	64.45	65.45	65.22	66.30	0.612	0.469
EM g/day	43.62	47.92	44.46	45.36	0.352	0.889
FCR-EM kg/kg	2.50	2.27	2.42	2.43	0.365	0.046
FCR/DZ kg/dz	1.94	1.79	1.91	1.93	0.381	0.035
BE %	2.18	2.47	2.59	2.47	0.950	0.236
DE %	1.75	2.85	2.72	2.85	0.276	0.292

⁎ EP: Egg production; MFI: Morning feed intake; AFI: Afternoon feed intake; TFI: Total feed intake; EW: Egg weight; EM: Egg mass; FCR-EM: Feed conversion ratio per egg mass; FCR/DZ: Feed conversion ratio per dozen eggs; BE: Broken eggs; DE: Dirty eggs. SEM: Standard error of the mean. CONTROL: Standard diet provided twice daily. formulated with 2.800 kcal/kg ME. 15.51% CP. and 4.55% Ca. LOW: Morning diet containing 5% more CP and ME and 40% less Ca than the control diet. and afternoon diet containing 5% less CP and ME and 40% more Ca. MODERATE: Morning diet containing 8% more CP. 10% more ME. and 50% less Ca. and afternoon diet containing 8% less CP. 10% less ME. and 50% more Ca. INTENSIVE: Morning diet containing 11% more CP. 15% more ME. and 60% less Ca. and afternoon diet containing 11% less CP. 15% less ME. and 60% more Ca. Different superscript letters within a row indicate significant differences according to Tukey’s test at the 5% probability level. SEM: Standard error of the mean.

Feed intake during the morning period showed a significant difference, with a tendency to increase as *split-feeding* intensity increased, suggesting that the treatments stimulated daytime intake by providing diets better aligned with the physiological requirements of this period. This response reinforces that modulation of feed supply throughout the day influences the ingestive behavior of laying hens, favoring greater intake when metabolic demand for albumen formation is higher, a process that occurs predominantly in the early hours after oviposition (Santos et al., 2017).

Conversely, feed intake during the afternoon period was also significantly affected (P = 0.0094), decreasing from 55.72 g/hen/day in the control group to 49.92 g/hen/day in the most intense *split-feeding* treatment. This reduction in afternoon intake suggests that the *split-feeding* strategy promoted greater efficiency in nutrient utilization, particularly calcium, which is offered in a more concentrated form during this period. During the late afternoon and nighttime, the eggshell calcification process begins in the uterus (Akter et al., 2025). As the experimental design was based on the temporal distribution of nutrients rather than differences in ingredient composition or physical characteristics of the diets, the observed intake is associated with nutrient allocation throughout the day.

To further clarify the nutrient intake dynamics between feeding periods, the intake of metabolizable energy, crude protein, and calcium was calculated for the morning and afternoon periods and is presented in Supplementary Table S1.

From a physiological standpoint, this result regarding feed intake indicates that the strategic provision of diets with higher calcium content in the afternoon reduced the need for volumetric intake without compromising plasma mineral availability. This occurs because intestinal calcium absorption depends not only on the ingested amount but also on the absorptive capacity of the intestinal tract, which is primarily regulated by the biologically active form of vitamin D₃. Parathyroid hormone stimulates the renal conversion of vitamin D₃ into 1,25-dihydroxycholecalciferol [1,25(OH)₂D₃], which increases the expression of proteins involved in transcellular calcium transport, thereby enhancing intestinal absorption efficiency in the hours following feeding (Lim and Ryu, 2022; Ribeiro et al., 2025).

From a physiological perspective, the reduced feed intake observed in the afternoon may be associated with the higher calcium concentration provided during this period, which coincides with the onset of eggshell formation. This alignment between nutrient supply and physiological demand may reduce the need for greater feed intake while maintaining adequate mineral availability for eggshell calcification.

These results are consistent with those reported by El-Razek et al. (2020), who observed lower feed intake in laying hens fed differently in the morning and afternoon without impairing productive performance. Similarly, Jahan et al. (2024) reported differences in feed utilization and waste when feed supply was adjusted according to calcium and energy requirements throughout the day, highlighting the influence of nutrient distribution on feeding patterns.

Egg weight was not affected by the treatments, indicating that feed fractionation did not alter egg size or the amount of mass produced per hen. This stability is desirable, as it demonstrates that split feeding allows optimization of nutrient intake without compromising productive yield. According to Tunç and Cufadar (2015), the temporal distribution of nutrients can improve calcium utilization for eggshell formation, although its effect on egg weight is generally small, which agrees with the results observed in the present study.

The percentages of broken and dirty eggs showed high variability, with no significant differences among treatments. This variability may be associated with environmental and management factors (humidity and collection frequency), which are known to critically affect shell quality and integrity more than feeding regimen (Ikusika et al., 2025). Therefore, split feeding did not compromise external egg quality, maintaining loss levels similar to the conventional system.

When analyzing egg quality data in Table 3, hens subjected to differentiated feed supply showed slight improvements in eggshell strength. Although the magnitude of this effect was relatively small, it may reflect improved synchronization between calcium supply and the period of eggshell formation. This response differs from traditional calcium dose–response studies, as it is associated with the temporal distribution of nutrients rather than an increase in total calcium intake. This finding reinforces the physiological importance of providing dietary calcium at the appropriate time, typically in the late afternoon, when the uterus initiates the eggshell calcification process (Saki et al., 2019). Thus, appropriate timing of calcium supply helps align mineral availability with the metabolic demand of the shell gland.

**Table 3:** Egg quality of laying hens fed different *split-feeding* strategies.

Variables*	**CONTROL**	**LOW**	**MODERATE**	**INTENSIVE**	***P-Value***	**SEM**
SR kgf	2.34b	2.66a	2.49ab	2.49ab	0.013	0.036
ST mm	0.354	0.342	0.327	0.342	0.089	0.004
SG g/cm³	1.0632	1.0753	1.0709	1.0706	0.535	0.003
SP %	9.72a	9.62ab	9.26b	9.40ab	0.007	0.052
AP %	65.68	66.06	65.60	65.88	0.676	0.143
YP %	24.60	24.32	25.14	24.72	0.152	0.127
HU	82.30bc	80.32c	85.06ab	85.55a	<0.001	0.455
YC	6.73	6.67	6.92	6.86	0.250	0.049
YI	0.379ab	0.370b	0.384a	0.371ab	0.031	0.002

⁎ SR: Shell resistance; ST: Shell thickness; SG: Specific gravity; SP: Shell percentage; AP: Albumen percentage; YP: Yolk percentage; HU: Haugh unit; YC: Yolk color. YI: Yolk index. SEM: Standard error of the mean. CONTROL: Standard diet provided twice daily, formulated with 2,800 kcal/kg ME, 15.51% CP, and 4.55% Ca. LOW: Morning diet containing 5% more CP and ME and 40% less Ca than the control diet, and afternoon diet containing 5% less CP and ME and 40% more Ca. MODERATE: Morning diet containing 8% more CP, 10% more ME, and 50% less Ca, and afternoon diet containing 8% less CP, 10% less ME, and 50% more Ca. INTENSIVE: Morning diet containing 11% more CP, 15% more ME, and 60% less Ca, and afternoon diet containing 11% less CP, 15% less ME, and 60% more Ca. Different superscript letters within a row indicate significant differences according to Tukey’s test at the 5% probability level.

The eggshell calcification process occurs predominantly during the late afternoon and nighttime and depends on adequate calcium availability during this period. In this context, the split-feeding strategy aims to better align calcium supply with the physiological demand for shell formation. However, considering that the observed effects on eggshell quality were relatively limited and not consistent across all treatments, these results should be interpreted with caution.

Although eggshell thickness and specific gravity did not show significant differences, mean values remained within the adequate range for commercial laying hens, as reported by Guo and Kim (2012), indicating that feed fractionation did not impair shell formation. Shell percentage showed a significant difference, being lower in the MODERATE treatment (9.26%) compared with the CONTROL treatment (9.72%). This response may be associated with differences in calcium intake distribution throughout the day (Supplementary Table S1), reflecting the temporal allocation of nutrients rather than a reduction in total calcium availability. This pattern suggests adjustments in mineral deposition without compromising shell resistance or structural integrity, since the LIGHT and INTENSE treatments did not differ from the CONTROL.

Haugh units were significantly influenced by the treatments, showing a marked increase in the MODERATE and INTENSE groups (85.06 and 85.55, respectively) compared with the CONTROL and LIGHT treatments. This response may be associated with differences in nutrient intake distribution throughout the day (Supplementary Table S1), particularly protein and amino acid supply, which are directly related to albumen synthesis. Values above 80 indicate excellent internal quality, suggesting that split feeding contributed to preserving albumen quality. According to Chang et al. (2024), high Haugh unit values are directly associated with greater albumen height and structural integrity of albumen proteins, especially ovomucin, whose degradation leads to albumen liquefaction and reduced Haugh units in older hens. In addition, Hwang et al. (2025) reported that laying hens subjected to split feeding with differentiated crude protein levels showed higher Haugh units, attributed to improved amino acid availability for protein synthesis in the magnum, the organ responsible for albumen protein secretion. Thus, the results of the present study suggest that nutritional balance throughout the day promoted greater protein stability and reduced albumen degradation, resulting in eggs with improved internal quality.

Yolk index was also significantly affected, with a higher value observed in the MODERATE treatment (0.384), indicating firmer yolks with a better height-to-diameter ratio. This response may be associated with a more balanced distribution of nutrient intake throughout the day (Supplementary Table S1), suggesting that intermediate split-feeding intensity may better align nutrient supply with yolk formation processes. According to Ji et al. (2023) and Réhault-Godbert et al. (2019), higher yolk index values are associated with greater vitelline membrane integrity and adequate lipid and protein deposition, which are determinant factors for yolk consistency and sphericity. In this context, a more synchronized supply of energy and protein throughout the day may have contributed to improved nutrient partitioning, reducing excessive substrate diversion to hepatic lipogenesis and promoting more balanced lipid deposition in the yolk, without necessarily affecting feed conversion ratio (Table 2). This metabolic adjustment contributes to maintaining yolk structure and higher yolk index values.

Conversely, albumen percentage, yolk percentage, and yolk color were not influenced, indicating that split feeding did not promote expressive changes in the internal distribution of egg components.

**Fig. 2:**
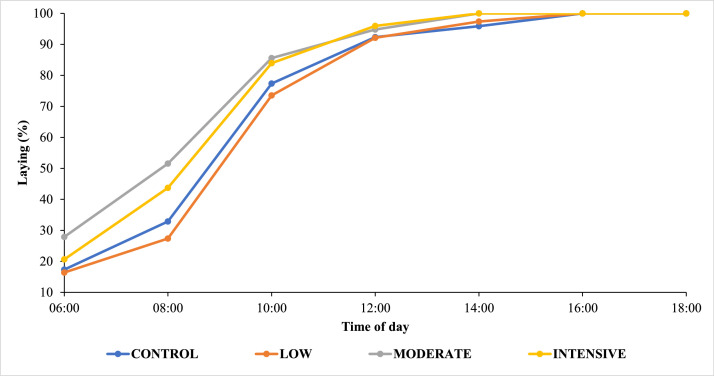
Laying time of hens fed different *split-feeding* strategies. CONTROL: Standard diet provided twice daily, formulated with 2,800 kcal/kg ME, 15.51% CP, and 4.55% Ca. LOW: Morning diet containing 5% more CP and ME and 40% less Ca than the control diet, and afternoon diet containing 5% less CP and ME and 40% more Ca. MODERATE: Morning diet containing 8% more CP, 10% more ME, and 50% less Ca, and afternoon diet containing 8% less CP, 10% less ME, and 50% more Ca. INTENSIVE: Morning diet containing 11% more CP, 15% more ME, and 60% less Ca, and afternoon diet containing 11% less CP, 15% less ME, and 60% more Ca.

The results shown in Fig. 2 indicate that hens subjected to moderate and intense split-feeding strategies exhibited a higher concentration of oviposition during the early hours of the day, reaching a peak at 14:00 h, whereas control hens reached peak oviposition only at 16:00 h. This pattern reinforces that fractionated nutrient supply throughout the day influences the physiological rhythm of oviposition, anticipating the laying time.

Although changes in oviposition timing could potentially influence egg formation dynamics, egg weight was not affected by the treatments (Table 2), indicating that the observed shift in laying rhythm was not sufficient to modify egg mass. This suggests that hens maintained stable egg formation processes despite temporal changes in oviposition.

From a practical perspective, split feeding can be a useful management tool to advance egg collection schedules. By providing energy- and protein-rich diets in the morning and calcium-rich diets in the afternoon, hen metabolism adjusts more efficiently to the oviposition cycle, favoring synchronization between egg formation in the oviduct and nutrient availability (De Los Mozos et al., 2014), resulting in most eggs being laid earlier. Consequently, egg collection can begin around 10:00 h, coinciding with the laying peak, which reduces egg residence time on conveyors, minimizes breakage and contamination, and optimizes labor efficiency (Tumová et al., 2009). Thus, synchronization between nutritional strategy and collection management allows greater productive and sanitary efficiency without negatively affecting overall laying performance.

Serum biochemical parameters indicated that split feeding did not disrupt the overall metabolic homeostasis of laying hens (Table 4), although significant differences were observed for some variables, such as urea, triglycerides, and cholesterol, reflecting specific metabolic adjustments.

**Table 4:** Serum biochemical parameters of laying hens fed different *split-feeding* strategies.

Variables*	**CONTROL**	**LOW**	**MODERATE**	**INTENSIVE**	***P-Value***	**SEM**
GLU mg/dL	209.13	208.13	218.63	204.25	0.312	2.766
CHOL mg/dL	121.25ab	158.63a	147.50ab	80.50b	0.031	10.437
TG mg/dL	1141.50ab	2268.13a	2057.75a	356.13b	0.006	230.145
UA mg/dL	2.70	2.44	2.10	1.92	0.544	0.202
U mg/dL	6.00ab	6.50a	4.87b	6.12ab	0.037	0.214
CRE mg/dL	0.56	0.53	0.52	0.51	0.390	0.010
AST U/L	273.50	282.63	284.63	257.25	0.769	9.828
ALT U/L	4.37	4.62	4.62	4.12	0.564	0.142
GGT U/L	35.75	38.50	35.00	39.50	0.328	0.992
ALP U/L	270.00	258.75	333.63	266.50	0.592	21.139
TP g/dL	5.36	5.65	5.12	4.74	0.176	0.151
ALB g/dl	2.36	2.54	2.43	2.27	0.130	0.041
Ca mg/dL	24.08	28.33	26.41	24.79	0.394	0.932
P mg/dL	4.33	5.33	4.32	4.26	0.244	0.216

⁎ GLU: Glucose; CHOL: Cholesterol; TG: Triglycerides; UA: Uric acid; U: Urea; CRE: Creatinine; AST: Aspartate aminotransferase; ALT: Alanine aminotransferase; GGT: Gamma-glutamyltransferase; ALP: Alkaline phosphatase; TP: Total protein; ALB: Albumin; Ca: Calcium; P: Phosphorus. SEM: Standard error of the mean. CONTROL: Standard diet provided twice daily, formulated with 2,800 kcal/kg ME, 15.51% CP, and 4.55% Ca. LOW: Morning diet containing 5% more CP and ME and 40% less Ca than the control diet, and afternoon diet containing 5% less CP and ME and 40% more Ca. MODERATE: Morning diet containing 8% more CP, 10% more ME, and 50% less Ca, and afternoon diet containing 8% less CP, 10% less ME, and 50% more Ca. INTENSIVE: Morning diet containing 11% more CP, 15% more ME, and 60% less Ca, and afternoon diet containing 11% less CP, 15% less ME, and 60% more Ca. Different superscript letters within a row indicate significant differences according to Tukey’s test at the 5% probability level.

No significant differences were observed for glucose, uric acid, creatinine, aspartate aminotransferase, alanine aminotransferase, gamma-glutamyl transferase, alkaline phosphatase, total proteins, albumin, calcium, and phosphorus, indicating the absence of hepatic overload or relevant metabolic imbalances among treatments.

Lipid metabolism parameters showed significant responses to feeding strategies. Cholesterol concentrations were higher in the LIGHT and MODERATE treatments (158.63 and 147.50 mg/dL) and decreased in the INTENSE treatment (80.50 mg/dL). The same pattern was observed for triglycerides, with values of 2268.13 and 2057.75 mg/dL in the LIGHT and MODERATE treatments, respectively, and a marked reduction to 356.13 mg/dL in the most fractionated treatment. This response may be associated with differences in nutrient intake distribution throughout the day (Supplementary Table S1), particularly metabolizable energy and protein supply, which are closely related to hepatic lipogenesis and lipoprotein synthesis.

According to Lima et al. (2025), laying hens typically present physiological cholesterol concentrations between 112.71 and 126.25 mg/dL and triglyceride concentrations between 1423.36 and 1753.66 mg/dL. Thus, the LIGHT and MODERATE groups remained within or slightly above the ideal range, whereas the INTENSE treatment showed lower-than-expected values, suggesting reduced hepatic lipogenesis and vitellogenic lipoprotein synthesis, possibly reflecting a more pronounced temporal redistribution of nutrients.

According to Faria et al. (2021), cholesterol and triglycerides are essential for avian energy and reproductive metabolism, participating in the formation of steroid hormones, bile acids, and yolk. Therefore, adequate levels of these metabolites reflect good hepatic function and proper lipid mobilization to reproductive tissues. The marked reduction observed in the INTENSE treatment may indicate a metabolic adjustment resulting from greater nutritional synchronization, with reduced stimulation of hepatic lipid synthesis, without necessarily implying immediate impairment of productive performance, but suggesting lower availability of lipid substrates for yolk deposition under conditions of higher physiological demand.

According to Faria et al. (2021), cholesterol and triglycerides are essential for avian energy and reproductive metabolism, participating in the formation of steroid hormones, bile acids, and yolk. Therefore, adequate levels of these metabolites reflect good hepatic function and proper lipid mobilization to reproductive tissues. The marked reduction observed in the INTENSE treatment may be associated with differences in nutrient intake distribution throughout the day (Supplementary Table S1), particularly in metabolizable energy and protein supply, which are directly related to hepatic lipogenesis and lipoprotein synthesis. In this context, a more pronounced temporal redistribution of nutrients may have reduced the stimulation of hepatic lipid synthesis, without necessarily implying immediate impairment of productive performance, but suggesting lower availability of lipid substrates for yolk deposition under conditions of higher physiological demand.

Urea showed a significant difference, with lower concentration in the MODERATE treatment (4.87 mg/dL), indicating improved utilization of dietary amino acids and reduced nitrogen excretion. This response may be associated with differences in nutrient intake distribution throughout the day (Supplementary Table S1), particularly protein supply.

The fact that this effect was observed only in the MODERATE treatment suggests a non-linear response, in which intermediate split-feeding intensity may have provided a more balanced synchronization between amino acid availability and metabolic demand, reducing excess amino acid catabolism. According to Faria et al. (2021), reduced urea levels are associated with greater protein efficiency and balanced hepatic metabolism, as nitrogen excretion in birds occurs predominantly in the form of uric acid. Thus, the lower concentration observed in the MODERATE treatment suggests improved nutrient utilization and reduced metabolic waste without compromising hepatic protein synthesis.

Morphometric analysis of the reproductive tract (Table 5) demonstrated that *split-feeding* strategy distinctly modulated the morphology of the magnum and uterus, reflecting functional adaptations associated with albumen secretion and calcium deposition, respectively.

**Table 5:** Morphometric analysis of the reproductive tract of laying hens fed different *split-feeding* strategies.

Variables*	**CONTROL**	**LOW**	**MODERATE**	**INTENSIVE**	***P-Value***	**SEM**
**Uterus**						
FH (µm)	2380.63	2555.62	2278.1	2375.22	0.210	46.40
FW (µm)	422.18	454.33	517.73	523.66	0.153	18.80
FA (µm²)	999,574b	1,158,817ab	1,040,946ab	1,302,707a	0.026	38987.89
**Magnum**						
FH (µm)	2341.17b	3111.61a	3087.39a	2570.42b	<0.001	66.62
FW (µm)	830.34c	1000.18a	972.19ab	880.67bc	0.001	16.71
FA (µm²)	1,937,027b	3,060,933a	3,005,852a	2,311,687b	<0.001	79821.47

FH: Fold height; FW: Fold width; FA: Fold area. SEM: Standard error of the mean. CONTROL: Standard diet provided twice daily, formulated with 2,800 kcal/kg ME, 15.51% CP, and 4.55% Ca. LOW: Morning diet containing 5% more CP and ME and 40% less Ca than the control diet, and afternoon diet containing 5% less CP and ME and 40% more Ca. MODERATE: Morning diet containing 8% more CP, 10% more ME, and 50% less Ca, and afternoon diet containing 8% less CP, 10% less ME, and 50% more Ca. INTENSIVE: Morning diet containing 11% more CP, 15% more ME, and 60% less Ca, and afternoon diet containing 11% less CP, 15% less ME, and 60% more Ca. Different superscript letters within a row indicate significant differences according to Tukey’s test at the 5% probability level.

In the magnum, the significant increase in fold height, width, and area observed in the LIGHT and MODERATE treatments indicates greater mucosal development and expansion of the secretory surface, favoring albumen protein synthesis and release. According to Silva et al. (2025), magnum morphology varies according to diet and production phase, reflecting physiological responses dependent on nutritional status and reproductive cycle. Thus, the results of the intermediate treatments suggest that moderate feed fractionation provided more stable metabolic conditions and sustained trophic stimulation of the glandular epithelium. In contrast, in the INTENSE treatment, the more pronounced temporal distribution of nutrient intake (Supplementary Table S1), particularly energy and protein, may have reduced the constancy of substrate supply required for maximal secretory activity throughout the day.

In the uterus, responsible for calcium carbonate and pigment deposition, a significant difference was observed only for fold area, with increasing values according to split-feeding intensity and the highest value recorded in the INTENSE treatment. Although fold height and width did not differ statistically, the increasing trend of these variables, combined with the expansion of total fold area, indicates mucosal reorganization and increased functional surface for calcium ion secretion and transport. Silva et al. (2025) reported that mineral-supplemented hens showed a greater number and height of uterine folds, which may favor mineral deposition and eggshell formation.

This result is consistent with the greater afternoon calcium supply provided by intense split feeding, coinciding with the period of highest demand for eggshell calcification, which occurs mainly during the scotophase (Pelícia et al., 2009). Additionally, although egg weight was not affected, the improved internal egg quality observed in some treatments (Table 3), particularly Haugh unit values, may suggest a broader influence of nutrient synchronization on egg formation processes, including albumen deposition.

**Fig. 3:**
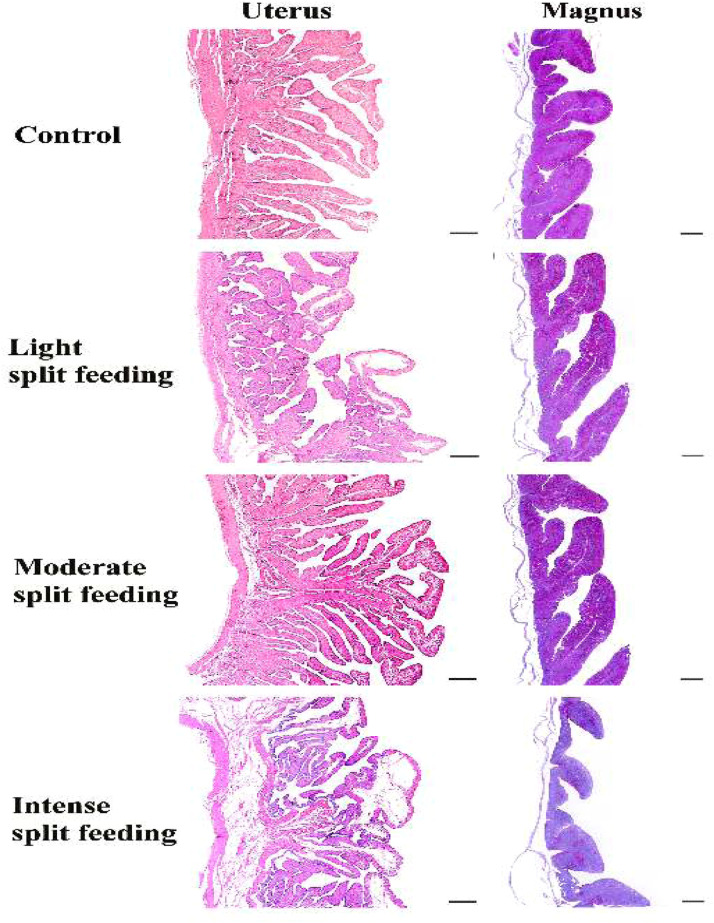
Photomicrographs of the uterus and magnum of semi-heavy laying hens fed under different intensities of split feeding during the period from 105 to 120 weeks of age. Light Split Feeding: morning diet with +5% of crude protein (CP) and metabolizable energy (ME) and –40% of Ca compared with the control diet; afternoon diet with –5% of CP and ME and +40% of Ca. Moderate Split Feeding: morning diet with +8% of CP, +10% of ME, and –50% of Ca; afternoon diet with –8% of CP, –10% of ME, and +50% of Ca. Intensive Split Feeding: morning diet with +11% of CP, +15% of ME, and –60% of Ca; afternoon diet with –11% of CP, –15% of ME, and +60% of Ca. Staining: PAS with hematoxylin. Scale bars: uterus and magnum 600 µm.

Histological evaluation of the reproductive tract evidenced structural differences consistent with the morphometric results observed (Fig. 3.). Overall, the morphological changes observed demonstrate that split feeding promoted organ-dependent responses in the reproductive tract of laying hens, reflecting specific adaptations to the functional demands of each segment.

The results presented in Table 6 indicate that *split-feeding* strategies influenced the Seedor index, especially in higher-intensity treatments, reflecting changes in the apparent density of the evaluated bones. The reduction in the Seedor index observed in the tibia and, more markedly, in the femur in the MODERATE and INTENSE treatments suggests adaptive adjustments in bone metabolism rather than an actual reduction in mineralization.

**Table 6:** Bone quality of laying hens fed different *split-feeding* strategies.

Variables*	**CONTROL**	**LOW**	**MODERATE**	**INTENSIVE**	***P-Value***	**SEM**
**Tibia**						
SI	106.63ab	110.76a	99.46b	97.48b	0.0055	1.6097
BS (kgf/cm²)	31.82	32.66	30.43	35.87	0.5785	1.3845
MM (%)	42.61	44.63	42.02	44.19	0.4358	0.6452
**Femur**						
SI	147.88a	133.75ab	119.58bc	115.50c	<0.0001	2.8928
BS (kgf/cm²)	27.26	32.08	26.50	28.23	0.3996	1.2293
MM (%)	44.92	49.47	46.27	49.78	0.0699	0.8017

⁎ SI: Seedor index; BS: Bone strength; MM: Mineral matter. SEM: Standard error of the mean. CONTROL: Standard diet provided twice daily, formulated with 2,800 kcal/kg ME, 15.51% CP, and 4.55% Ca. LOW: Morning diet containing 5% more CP and ME and 40% less Ca than the control diet, and afternoon diet containing 5% less CP and ME and 40% more Ca. MODERATE: Morning diet containing 8% more CP, 10% more ME, and 50% less Ca, and afternoon diet containing 8% less CP, 10% less ME, and 50% more Ca. INTENSIVE: Morning diet containing 11% more CP, 15% more ME, and 60% less Ca, and afternoon diet containing 11% less CP, 15% less ME, and 60% more Ca. Different superscript letters within a row indicate significant differences according to Tukey’s test at the 5% probability level.

This behavior is related to the physiological dynamics of calcium metabolism in laying hens, in which medullary bone acts as a temporary reserve to meet the high mineral demand during eggshell formation. According to Nascimento et al. (2025), the success of split feeding depends on adequate balancing of calcium, phosphorus, energy, and protein throughout the day, favoring controlled mobilization of medullary calcium without compromising structural bone.

Despite the reduction in the Seedor index, no significant differences were observed in the mechanical strength of the tibia and femur or in mineral matter content (ash), indicating that structural integrity and bone mineral content were preserved. As described by Khanal et al. (2020), maintenance of bone strength despite variations in apparent density indicates reorganization of bone microarchitecture and preservation of the collagen matrix, characteristics of physiological bone remodeling.

Overall, the results indicate that split feeding promoted bone adaptations compatible with the metabolic demands of egg production, without compromising skeletal health. These effects appear to be related to the temporal distribution of nutrients, particularly metabolizable energy, calcium, and protein, within the split-feeding program. Moderate-intensity treatments favored a balanced mobilization and replacement of minerals, whereas more intense treatments induced more pronounced changes in apparent bone density, likely reflecting adjustments in nutrient timing and availability. Importantly, these alterations occurred without impairing bone strength or overall mineralization, suggesting that strategic nutrient allocation throughout the day supports physiological bone remodeling in laying hens.

## Conclusion

Moderate-intensity split feeding, with a morning diet containing 3,080 kcal/kg of metabolizable energy, 16.70% crude protein, and 2.28% calcium, and an afternoon diet containing 2,520 kcal/kg of metabolizable energy, 14.32% crude protein, and 6.83% calcium, proved to be the most effective strategy for semi-heavy laying hens between 105 and 120 weeks of age. This feeding approach enhanced synchronization between nutrient supply and physiological demands, supporting bone adaptations, optimizing albumen and yolk deposition, and maintaining internal and external egg quality. The results indicate that temporal distribution of multiple nutrients rather than calcium supplementation alone can improve metabolic efficiency and promote physiological bone remodeling without compromising skeletal integrity or overall performance.

## CRediT authorship contribution statement

**Aline Beatriz Rodrigues:** Writing – review & editing, Writing – original draft, Methodology, Investigation, Formal analysis, Data curation, Conceptualization. **Adiel Vieira de Lima:** Writing – review & editing, Investigation, Formal analysis, Data curation, Conceptualization. **Paloma Eduarda Lopes de Souza:** Investigation, Formal analysis, Data curation, Conceptualization. **Carlos Henrique do Nascimento:** Investigation, Formal analysis, Data curation, Conceptualization. **Raiane dos Santos Silva:** Formal analysis, Data curation, Conceptualization. **Raul da Cunha Lima Neto:** Formal analysis, Data curation, Conceptualization. **Matheus Ramalho de Lima:** Formal analysis, Data curation, Conceptualization. **Apolônio Gomes Ribeiro:** Writing – review & editing, Formal analysis, Data curation, Conceptualization. **Edijanio Galdino da Silva:** Formal analysis, Data curation, Conceptualization. **Ariolino Moura de Oliveira Neto:** Formal analysis, Data curation. **Marcos Rafael de Sousa Rodrigues:** Formal analysis, Data curation, Conceptualization. **José de Arimatéia de Freitas Pinto:** Formal analysis, Data curation, Conceptualization. **Ricardo Romão Guerra:** Writing – review & editing, Methodology, Formal analysis, Data curation, Conceptualization. **Lucas Rannier Ribeiro Antonino Carvalho:** Writing – review & editing, Formal analysis, Data curation, Conceptualization. **Fernando Guilherme Perazzo Costa:** Writing – review & editing, Writing – original draft, Visualization, Validation, Supervision, Resources, Project administration, Methodology, Investigation, Funding acquisition, Formal analysis, Data curation, Conceptualization.

## Disclosures

The authors declare that they have no other conflicts of interest.

## Appendix Supplementary materials

Image, application 1

## Footnotes

Supplementary material associated with this article can be found, in the online version, at doi:10.1016/j.psj.2026.106917.
