# Supplementary material for: Split Feeding for semi-heavy laying hens from 105 to 120 weeks of age: Performance, egg quality, biochemical parameters, reproductive tract morphometry and bone quality
Source: Poult Sci. 2026 Apr 9;105(7):106917. doi: 10.1016/j.psj.2026.106917 (PMC13122803; doi:10.1016/j.psj.2026.106917)
Supplement: Supplementary file 1 [file mmc1.docx]

Supplementary Table S1. Nutrient intake of semi-heavy laying hens during morning and afternoon feeding periods under the split-feeding system

| **Variables** | **CONTROL** | **LOW** | **MODERATE** | **INTENSIVE** | ***P-Value*** | **SEM** | **CV (%)** |
| --- | --- | --- | --- | --- | --- | --- | --- |
| ME^1^ kcal/hen/day | 149.85b | 158.04b | 173.85a | 186.84a | <0.0001 | 3.6000 | 4.39 |
| CP^1^ g/hen/day | 8.30c | 8.78bc | 9.43ab | 10.01a | <0.0001 | 0.1700 | 4.38 |
| Ca^1^ g/hen/day | 2.43a | 1.47b | 1.28c | 1.06d | <0.0001 | 0.1200 | 4.58 |
| ME^2^ kcal/hen/day | 156.01a | 141.33b | 129.78c | 118.81c | <0.0001 | 3.4100 | 4.55 |
| CP^2^ g/hen/day | 8.64a | 7.79b | 7.37bc | 6.86c | <0.0001 | 0.1600 | 4.54 |
| Ca^2^ g/hen/day | 2.53b | 3.39a | 3.52a | 3.64a | <0.0001 | 0.1000 | 4.55 |

^1^ Morning; ^2^ Afternoon; ME: Metabolizable energy; CP: Crude protein; Ca: Calcium.
